# Diversity among multidrug-resistant enterococci.

**DOI:** 10.3201/eid0401.980106

**Published:** 1998

**Authors:** B E Murray

**Affiliations:** University of Texas Houston-Medical School, Houston 77030, USA.

## Abstract

Enterococci are associated with both community- and hospital-acquired infections. Even though they do not cause severe systemic inflammatory responses, such as septic shock, enterococci present a therapeutic challenge because of their resistance to a vast array of antimicrobial drugs, including cell-wall active agents, all commercially available aminoglycosides, penicillin and ampicillin, and vancomycin. The combination of the latter two occurs disproportionately in strains resistant to many other antimicrobial drugs. The propensity of enterococci to acquire resistance may relate to their ability to participate in various forms of conjugation, which can result in the spread of genes as part of conjugative transposons, pheromone-responsive plasmids, or broad host-range plasmids. Enterococcal hardiness likely adds to resistance by facilitating survival in the environment (and thus enhancing potential spread from person to person) of a multidrug-resistant clone. The combination of these attributes within the genus Enterococcus suggests that these bacteria and their resistance to antimicrobial drugs will continue to pose a challenge.


### Diversity among Multidrug-Resistant Enterococci

**Barbara E. Murray, M.D.**
University of Texas Houston-Medical School, Houston, Texas, USA

Enterococci are associated with both community- and hospital-acquired infections. Even though they do not cause severe systemic inflammatory responses, such as septic shock, enterococci present a therapeutic challenge because of their resistance to a vast array of antimicrobial drugs, including cell-wall active agents, all commercially available aminoglycosides, penicillin and ampicillin, and vancomycin. The combination of the latter two occurs disproportionately in strains resistant to many other antimicrobial drugs. The propensity of enterococci to acquire resistance may relate to their ability to participate in various forms of conjugation, which can result in the spread of genes as part of conjugative transposons, pheromone-responsive plasmids, or broad host-range plasmids. Enterococcal hardiness likely adds to resistance by facilitating survival in the environment of a multidrug-resistant clone, thus enhancing potential spread from person to person. The combination of these attributes within the genus *Enterococcus* suggests that these bacteria and their resistance to antimicrobial drugs will continue to pose a challenge.

Enterococci, which have been known as a cause of infective endocarditis for close to a century, more recently have been recognized as a cause of nosocomial infection and "superinfection" in patients receiving antimicrobial agents (1). The enterococcus is now receiving increased attention because of its resistance to multiple antimicrobial drugs, which probably explains a large part of its prominence in nosocomial infections. The most common enterococci-associated nosocomial infections are infections of the urinary tract, followed by surgical wound infections and bacteremia (1-3). Enterococci are often present in intraabdominal and pelvic infections, although not all patients with such infections require specific antienterococcal therapy. Other enterococcal infections include infections (including meningitis and bacteremia) in very ill neonates; central nervous system infections in adults, typically with a history of central nervous system surgery or intrathecal chemotherapy; and rarely, osteomyelitis and pulmonary infections. Enterococci frequently arise from colonization of indwelling T tubes, causing liver or biliary infection in liver transplant patients (1).

Address for correspondence: Barbara E. Murray, Division of Infectious Diseases, University of Texas Houston-Medical School, 6431 Fannin, 1.728 JFB, Houston, TX 77030, USA; fax: 713-500-6761; e-mail: iminfdis@heart.med.uth.tmc.edu.

### Antimicrobial Resistance

Most enterococci have naturally occurring or inherent resistance to various drugs, including cephalosporins and the semisynthetic penicillinase-resistant penicillins (e.g., oxacillin) and clinically achievable concentrations of clindamycin and aminoglycosides. Compared with streptococci, most enterococci are relatively resistant to penicillin, ampicillin, and the ureidopenicillins, with MICs of 1 µg/ml to 8 µg/ml for most *Enterococcus faecalis* and even higher for most *E. faecium*. Many enterococci are also tolerant to the killing effects of cell-wall active agents, including ampicillin and vancomycin; recent data suggest that this property may not be inherent, but rather acquired after exposure to antibiotics (4). Inherent in vivo resistance of *E. faecalis* to trimethoprim-sulfamethoxazole may explain the lack of efficacy in animal models. In vitro, trimethoprim-sulfamethoxazole readily inhibits most enterococci at low concentrations, but this activity is lessened by exogenous folates (5). Moreover, bactericidal activity against *E. faecalis* seems unreliable and very method dependent (6). In animal models, this combination has not shown good activity and is not generally accepted as an effective antienterococcal therapy, especially for systemic infections (7,8).

In addition to natural resistance to many agents, enterococci have also developed plasmid- and transposon-mediated resistance to tetracycline (as well as minocycline and doxycycline), erythromycin (plus the newer compounds azithromycin and clarithromycin), chloramphenicol, high levels of trimethoprim, and high levels of clindamycin.

The propensity of *E. faecalis* to acquire multiple antibiotic-resistance traits may result from a variety of distinctly different mechanisms for conjugation, i.e., bacterial mating. The best studied system of conjugation involves oligopeptides called pheromones and pheromone-responsive plasmids (9; Figure 1). Briefly, strains of *E. faecalis* typically secrete into the culture medium a number of different small peptide sex pheromones specific for different types of plasmids. When a cell containing a pheromone-responsive plasmid (the potential donor cell) comes into contact with its corresponding pheromone, transcription of a gene on the plasmid is turned on, resulting in the synthesis of a sticky substance (called aggregation substance) on its surface. When the donor cell bumps into another *E. faecalis*, aggregation substance, which contains two Arg-Gly-Asp motifs, sticks to the binding substance on the surface of most *E. faecalis* cells, causing them to clump together. In the test tube, clumps of cells actually fall to the bottom of the tube, resulting in a visible aggregate. By a process not yet well understood, the pheromone-responsive plasmid can then transfer from the donor bacterium to the other (recipient) bacterium. Once the recipient cell has acquired this particular plasmid, the synthesis of the corresponding sex pheromone is shut off to prevent self-clumping. This system of conjugation, which occurs primarily in *E. faecalis,* is highly efficient and results in transfer of plasmids in both filter and broth matings.

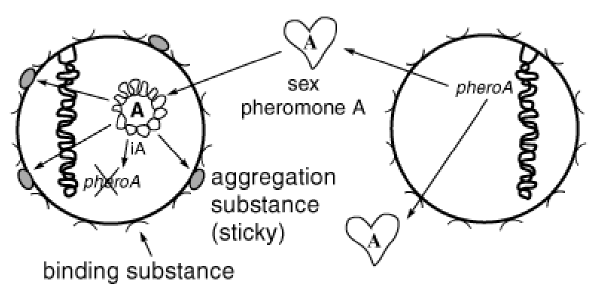

Figure 1. *Enterococcus faecalis* pheromone-responsive conjugative system.
Pheromone A released from the potential recipient cell (right) interacts with plasmid A in the potential donor cell (left) to induce synthesis of aggregation substance. Attachment of aggregation substance to binding substance causes the cells to clump into visible aggregates. Once the pheromone-responsive plasmid A has transferred from donor to recipient cell, synthesis of pheromone A is shut off.

Another system of conjugation, also not well understood, involves broad host-range plasmids that can transfer among species of enterococci and other gram-positive organisms such as streptococci and staphylococci (10). The transfer frequency is generally much lower than with the pheromone system and is much more efficient with filter than with broth matings. Since staphylococci, streptococci, and enterococci share a number of resistance genes, these broad host-range plasmids may be a mechanism by which some of these resistance genes have spread among different genera.

A third type of conjugation, which involves conjugative transposons, may also explain the spread of resistance genes to many different species (11). As opposed to ordinary transposons, which can jump within a cell from one DNA location to another, conjugative transposons also encode the ability to bring about conjugation between different bacterial cells. Since plasmids typically require rather complex machinery for replication (often depending on successful interactions with host proteins) and must face additional problems of surface exclusion and incompatibility, conjugative transposons (which do not replicate, but instead insert into the chromosome or into a plasmid of the new host) appear to be an even more efficient and far-reaching way of disseminating a resistance gene. This may explain why the *tetM* gene of the conjugative transposon Tn*916* has spread beyond the gram-positive species into gram-negative organisms, including gonococci, meningococci, and *Haemophilus ducreyi,* as well as into mycoplasma and ureaplasma, among others (12,13). Other resistance genes, including those encoding resistance to erythromycin and kanamycin, are also found on conjugative transposons; these frequently contain or are related to Tn*916*. Such transposons may have evolved from a Tn*916* ancestor; their emergence suggests the possibility of further dissemination of resistance among gram-positive organisms. Particularly ominous are reports of the *vanB* gene cluster within large conjugative chromosomal elements that appear similar, at least in function, to conjugative transposons (14).

### High-Level Aminoglycoside Resistance

Although some acquired resistance of enterococci is not clinically important because the agents involved are not commonly used, other resistance greatly affects enterococcal therapy; high-level resistance (HLR) to aminoglycosides is an example. This resistance is added onto the normal low-level resistance of enterococci to aminoglycosides and typically results in MICs of $\geq$ 2,000 µg/ml. This degree of resistance predicts, without exception, resistance to synergism between cell-wall active agents and the aminoglycoside to which the organism is highly resistant (1). High-level aminoglycoside resistance is most often due to aminoglycoside-modifying enzymes; HLR to streptomycin can also be ribosomal, that is, due to a mutation that results in ribosomes resistant to streptomycin inhibition. HLR to kanamycin (without gentamicin) is a fairly common trait and is due to the production of a 3'-phosphotransferase, APH(3')-III. This enzyme is important because it also eliminates synergism between cell-wall active agents and amikacin (through phosphorylation of the 3'-hydroxyl group), although it does not necessarily confer HLR to amikacin. HLR to gentamicin results from the bifunctional protein (AAC(6')-I/APH(2")-I), encoded by a single gene with two active sites, one with 6'-acetyltransferase activity and the other, 2"-phosphotransferase activity (15). The combination of these activities results in HLR or resistance to synergism for all commercially available aminoglycosides except streptomycin, which is not modified by this enzyme. However, HLR to streptomycin (due to either ribosomal resistance or a streptomycin adenylyltransferase) is also common and can coexist with the gene(s) for HLR to other aminoglycosides. Spectinomycin is also not modified by the bifunctional enzyme, but this agent, which is not a true aminoglycoside, is not generally bactericidal against enterococci and does not appear to show synergism with cell-wall active agents.

Strains of enterococci from patients with endocarditis and other serious infections for whom combination therapy is desired should be screened for HLR to streptomycin and gentamicin. HLR screening for tobramycin is not generally performed or advisable It could, in principle, be used for *E. faecalis*, but *E. faecium* isolates have a chromosomally encoded, naturally occurring gene for a 6'-acetyltransferase that eliminates synergism with tobramycin, although it does not cause HLR (MICs are typically 128-500 µg/ml); a probe for this gene has been used to confirm the identification of *E. faecium* isolates to species. In addition, HLR of an *E. faecium* isolate (without HLR to gentamicin) to tobramycin, due to an adenylyltransferase, was recently described (16). Therefore, the use of tobramycin for possible synergism in serious enterococcal infections would need to be preceded by screening for HLR to tobramycin (a test that is not commonly available), as well as for identification to species, neither of which is practical.

More recently, veterinary and human isolates resistant to moderate levels of gentamicin (256 µg/ml) were found to have a new gentamicin-modifying enzyme encoded by a gene designated *aph(2")-Ic* (17). This gene conferred resistance to synergism between gentamicin and cell-wall active agents and may be less easily detected than strains producing the bifunctional enzyme.

### Beta-Lactamase– and non-Beta-Lactamase–Associated Penicillin Resistance

The first known penicillinase-producing isolate of enterococcus was an isolate of *E. faecalis* recovered from a patient in Houston, Texas, in 1981 (18). Although rare, these isolates have been reported from the United States (Texas, Florida, North Carolina, Delaware, Pennsylvania, New York, Massachusetts, and Connecticut), Lebanon, Canada, and Argentina (19). Like other enterococci, beta-lactamase-producing strains have been found as colonizers, as in the large “outbreak” of colonization in a Boston children’s hospital, but they have also been associated with true infections, as was demonstrated by cases at a Virginia Veterans Administration hospital, by isolates from Argentina, and in other reports (20,21). The enterococcal penicillinase gene, identical to the gene encoding staphylococcal type A penicillinase, almost always occurs in strains with HLR to gentamicin and is often found on a transferable plasmid that also contains *aph(2")-Ia/aac(6')-Ie*. The relatively low levels of beta-lactamase produced by enterococci result in a marked inoculum effect with these strains so that at low and even moderate inocula ($10^3$-$10^5$ CFU/ml), penicillinase-producing enterococci usually appear no more resistant than other enterococci, while at high inocula ($\geq 10^7$ CFU/ml), these organisms are usually highly resistant to penicillin, ampicillin, and ureidopenicillins. The activity of the penicillinase is reversed by the beta-lactamase inhibitors clavulanate, sulbactam, and tazobactam; in animal models of endocarditis, beta-lactamase inhibitors have been shown to markedly enhance the therapeutic efficacy of ampicillin or penicillin. In the clinical laboratory, penicillinase-producing enterococci are generally not detected by routine laboratory susceptibility testing, such as MICs or disk diffusion. For this reason, if a penicillin is to be used for therapy, enterococcal isolates from patients with endocarditis or other serious infections should be tested for penicillinase production by using a specific beta-lactamase test such as the chromogenic cephalosporin nitrocefin.

Nonpenicillinase-producing, penicillin-resistant enterococci have been reported for decades and usually are *E. faecium*. Until recently, MICs of penicillin typically ranged from 8 µg/ml to 64 µg/ml, with an occasional isolate having higher levels of resistance. However, increasingly, strains with much higher levels of penicillin resistance have been reported (22). Whether a large number of strains have converted from low-level to high-level resistance or a more limited number of strains have been disseminated is unclear. The mechanisms involved in this resistance are overproduction of a low-affinity penicillin-binding protein (a cell-wall synthesis enzyme) and a further decrease in the affinity of one of these enzymes for penicillin (23). As a possible explanation for why many vancomycin-resistant *E. faecium* also have very high levels of resistance to ampicillin, Rice and colleagues (24) showed that transfer of vancomycin resistance from one strain to another was linked to transfer of ampicillin resistance.

### Vancomycin Resistance

Most surprising in recent years has been the emergence among enterococci of acquired resistance to vancomycin. Vancomycin had been in clinical use since the 1950s, although it was not heavily used until the late 1970s and particularly the 1980s. Because multiple genes are involved in generating vancomycin resistance, the development of resistance was neither easy nor recent. Three phenotypes of vancomycin resistance (types A, B, and C) are now well described; a fourth, type D, has been recently reported (25). VanA-type strains are typically highly resistant to vancomycin and moderately to highly resistant to teicoplanin. This phenotype is often plasmid or transposon mediated and is inducible (i.e., exposure of bacteria to vancomycin results in the induction of the synthesis of several proteins that together confer resistance) (26).

In vancomycin-susceptible enterococci, D-alanyl-D-alanine (formed by an endogenous D-alanine-D-alanine ligase) is added to a tripeptide precursor to form a pentapeptide precursor. The D-Ala-D-Ala terminus is the target of vancomycin; once vancomycin has bound, the use of this pentapeptide precursor for further cell-wall synthesis is prevented. In the VanA phenotype, one of the proteins whose synthesis is induced by exposure of bacterial cells to vancomycin is called VanA; VanA is a ligase and resembles the D-alanine-D-alanine ligase from *Escherichia coli* and other organisms, including vancomycin-susceptible enterococci (27). VanA generates D-Ala-D-X, where X is usually lactate; the formation of D-lactate is due to the presence of VanH, a dehydrogenase encoded by *vanH*. The depsipeptide moiety, D-Ala-D-Lac, is then added to a tripeptide precursor, resulting in a depsipentapeptide precursor. Vancomycin does not bind to the D-Ala-D-Lac terminus, so this depsipentapeptide can be used in the remaining steps of cell-wall synthesis. However, when the normal pentapeptide precursor ending in D-Ala-D-Ala is also present, cells are not fully vancomycin resistant, despite the presence of D-Ala-D-Lac containing precursors. This apparent problem is taken care of in large part by *vanX*, which encodes a dipeptidase, VanX, that cleaves D-Ala-D-Ala, preventing its addition to the tripeptide precursor. Should any D-Ala-D-Ala escape cleavage and result in a normal pentapeptide precursor, *vanY* encodes an ancillary or back-up function. That is, it codes for a carboxypeptidase, VanY, which cleaves D-alanine and D-lactate from D-Ala-D-Ala and D-Ala-D-Lac termini, respectively, resulting in tetrapeptide precursors, to which vancomycin does not bind. The other genes involved in the VanA resistance complex include *vanR* and *vanS*, whose encoded proteins are involved in somehow sensing the presence of extracellular vancomycin or its effect and signaling intracellularly to activate transcription of *vanH*, *vanA*, and *vanX* (27). A final gene in the *vanA* cluster is *vanZ,* which encodes VanZ, the role of which is not known.

VanB, encoded by *vanB* in the *vanB* gene cluster, is also a ligase that stimulates the formation of D-Ala-D-Lac. The VanB phenotype is typically associated with moderate to high levels of vancomycin resistance but is without resistance to teicoplanin. This is explained by the observation that vancomycin, but not teicoplanin, can induce the synthesis of VanB and of $VanH_B$ and $VanX_B$. However, because mutants resistant to teicoplanin can readily be selected from VanB strains on teicoplanin-containing agar, clinical resistance would likely occur among VanB strains if teicoplanin were widely used. Most of the proteins encoded by the *vanA* gene cluster have homologues encoded by the *vanB* gene cluster, except for VanZ. The *vanB* gene cluster has an additional gene, *vanW*, of unknown function.

The VanC phenotype (low-level resistance to vancomycin, susceptible to teicoplanin) is an inherent (naturally occurring) property of *E. gallinarum* and *E. casseliflavus*. This property is not transferable and is related to the presence of species-specific genes *vanC-1* and *vanC-2*, respectively (28); a third possible species, *E. flavescens* and its gene *vanC-3*, are so closely related to *E. casseliflavus* and *vanC-2* that different names are probably not warranted (29). These species appear to have two ligases; the cell-wall pentapeptide, at least in *E. gallinarum*, ends in a mix of D-Ala-D-Ala and D-Ala-D-Ser (29,30). The genes *vanC-1* and *vanC-2* apparently lead to the formation of D-Ala-D-Ser containing cell-wall precursors, while D-Ala-D-Ala ligases, also present in these organisms, result in D-Ala-D-Ala. The presence of both D-Ala-D-Ala and D-Ala-D-Ser precursors may explain why many isolates of these species test susceptible to vancomycin and why even those isolates with decreased susceptibility display only low-level resistance.

VanD-type glycopeptide resistance has been recently described in an *E. faecium* isolate from the United States (25). The organism was constitutively resistant to vancomycin (MIC ≥ 64 µg/ml) and to low levels (4 µg/ml) of teicoplanin. Following polymerase chain reaction amplification with primers that amplify many D-Ala-D-Ala ligases, a 605-bp fragment was identified whose deduced amino acid sequence showed 69% identity to VanA and VanB and 43% identify to VanC.

### Molecular Epidemiology of Newer Resistance Traits

### High-Level Gentamicin Resistance

The DNA sequence of the gene encoding HLR to gentamicin in *E. faecalis* is the same as the sequence of the gentamicin resistance gene of staphylococci (15). Since this gene was well established in staphylococci by the 1970s but HLR to gentamicin was not reported in enterococci until 1979, the seemingly obvious conclusion is that this gene spread from staphylococci to enterococci rather than vice versa or, at least, staphylococci acquired it first. However, the disk diffusion method used in the 1970s and microtiter dilution MICs done later are capable of detecting gentamicin resistance in staphylococci but do not distinguish enterococci with high-level gentamicin resistance from those with low-level, inherent resistance. Therefore, since laboratories were not screening enterococci by special techniques for high-level gentamicin resistance, it cannot be definitively stated that this resistance did not appear in enterococci earlier or coincident with its emergence in staphylococci. However, several observations support the likelihood that gentamicin resistance appeared and disseminated in staphylococci before it did in enterococci. In 1971, Moellering et al. reported the lack of high-level gentamicin resistance among enterococci (31). Watanakunakorn reported the absence of high-level gentamicin resistance among 126 enterococci from 1980 to 1984, with HLR subsequently appearing in 1985 (32). Phillips et al. from the United Kingdom reported no highly gentamicin-resistant strains in 1969 to 1979 or 1980 to 1985 and appearance of strains in 1986 (33). Zervos et al. reported that only one (0.04%) of 269 isolates of *E. faecalis* had high-level gentamicin resistance in 1981; this figure gradually increased to 7.7% in 1984 (34). High-level gentamicin resistance in *E. faecium* appears to have occurred after its appearance in *E. faecalis*, with the first report occurring in 1988 (35).

Delineation of the molecular epidemiology of strains of enterococci was limited in the past by the lack of an easy, reliable, and widely accessible method for subspecies strain differentiation. Zervos and Schaberg reported the use of plasmid patterns in enterococci to suggest the intrahospital spread of strains with high-level gentamicin resistance. Pulsed-field gel electrophoresis (PFGE)

of *E. faecalis* with HLR to gentamicin found that different isolates from both the same and different locations had markedly different restriction endonuclease digestion patterns. That is, it found no evidence of a common strain or strains that predominated among gentamicin-resistant organisms (36). Strains isolated between 1981 and 1984 at the University of Michigan demonstrated that plasmids encoding high-level gentamicin resistance were heterogeneous, which again argues against clonal dissemination of a limited number of strains or plasmids to account for spread of this property (34,37). We have subsequently shown that gentamicin resistance in enterococci can be encoded on a transposon identical to that in staphylococci (38). In addition, the enterococcal gentamicin-resistance gene has been found in other genetic settings, one of which has also been found in North American *Staphylococcus aureus* with gentamicin resistance (39). Since all enterococci with HLR to gentamicin (MIC ≥ 2,000 µg/ml) that have been tested have hybridized with the same gene probe, this property could be termed a "gene epidemic." However, by the time gentamicin resistance was discovered in enterococci, this gene was already widespread with no evidence of either a common plasmid or a common or predominant strain.

Other genetic elements encoding HLR to gentamicin have also been described. Thal et al. have described a 27-kb element designated Tn*924* that encodes HLR to gentamicin and could be mobilized from the chromosome of an *E. faecalis* by a coresident plasmid (40). Rice et al. have described a large (ca. 60 kb) transferable element, tentatively named Tn*5385*, which appears to contain within it an 18-kb conjugative transposon (Tn*5381*) encoding tetracycline resistance and a 26-kb IS*256*-based composite transposon (Tn*5384*) encoding resistance to gentamicin and to erythromycin (41).

### Penicillin-Resistant Penicillinase-Producing Enterococci

PFGE analyses of penicillinase-producing enterococci have shown that a common penicillinase-producing strain (or "clone"), defined as having an identical or related chromosomal digestion pattern, was present in Texas, Florida, North Carolina (unpub. observation), Delaware, Pennsylvania, and Virginia, which had a large outbreak with numerous infections (42,43). Moreover, at each of these locations, all isolates of penicillinase-producing *E. faecalis* examined were derivatives of this strain; in the hospital in which this strain has been endemic for many years, a single penicillinase-producing isolate of *E. faecium*, the only such isolate ever reported, was also found (44). Among numerous non-Bla+ *E. faecalis*, a PFGE pattern similar to that of Bla+ *E. faecalis* has been found only once from an isolate from a Philadelphia hospital that had one of the Bla+ isolates (43 and unpub. obs.). Penicillinase-producing isolates from Connecticut, Boston, Canada (unpub. observations), Lebanon, and Argentina represent different strains (19). However, clonal relatedness of three isolates with an almost identical pattern was demonstrated in Connecticut (despite one of these lacking HLR to gentamicin) (45), and all six isolates in Argentina appeared to be a single strain. The Boston isolates have not been studied by PFGE, but a number of isolates from the same hospital had a single shared plasmid, suggesting that these also represent clonal dissemination of a single strain. While all of these penicillinase-producing *E. faecalis* could be detected by nitrocefin, a vancomycin-resistant *E. faecalis* that tested negative for penicillinase by nitrocefin but showed a marked inoculum effect with penicillin and destroyed penicillin in a bioassay has been reported (46). Thus, in contrast to high-level gentamicin resistance in enterococci, which appeared to be widely disseminated on different plasmids and in different strains by the time this phenotype was studied, penicillinase production in enterococci is still largely associated with a limited number of strains; moreover, in locations known to have more than one isolate, oligoclonal spread within each setting remains the rule.

### Nonpenicillinase-Producing Penicillin-Resistant Strains

PFGE analyses of highly penicillin-resistant *E. faecium* from the Medical Colleges of Virginia and Pennsylvania have shown that within each location, most highly resistant strains represented a single clone (47). Analysis of the PFGE patterns also raised the possibility, on the basis of similarities between patterns of isolates in these two different locations, that a strain may have spread from one institution to the other (47); this conclusion was supported by the finding that these isolates belonged to the same multilocus enzyme cluster (unpub. observations). Circumstantial evidence of intrahospital spread of highly penicillin-resistant *E. faecium* also comes from the study of Grayson et al. (22), who reported a sudden increase in more highly penicillin-resistant isolates of *E. faecium* at the Massachusetts General Hospital in 1988. Although no genetic analysis was done, the fact that gentamicin resistance also simultaneously appeared and most of the *E. faecium* highly resistant to penicillin were also highly resistant to gentamicin suggests clonal dissemination of one or a few strains within that hospital (22).

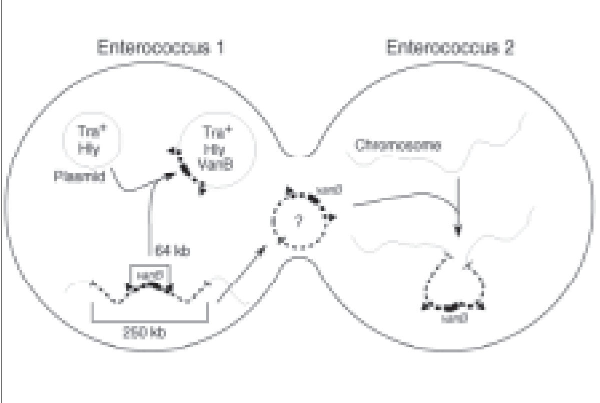

Figure 2. Potential modes of spread of vancomycin-resistant genes. Adapted in part from Quintiliani and Courvalin (14).
The *vanB* gene cluster (shown on the left) on a 64-kb transposon is part of a 250-kb mobile element shown to move from the chromosome of one enterococcus and insert into the chromosome of another. Although not demonstrated, circularization of the *vanB* containing large mobile elements resembles the mechanism described for conjugative transposons that can excise from the chromosome of one strain, circularize, transfer from one enterococcus to another, and reinsert into the chromosome of the recipient (such as the one on the right). The 64-kb transposon shown on the left can also jump to another plasmid within the host enterococcus. If it is a conjugative (Tra+) plasmid, that plasmid can then transfer by conjugation to other bacteria, taking the *van* resistance genes with it. In one instance, the vancomycin resistance transposon was shown to transpose to a plasmid encoding the virulence factor hemolysin (Hly).

### Vancomycin Resistance

Vancomycin resistance in enterococci is heterogeneous on many levels. For example, three different, well-described types of vancomycin resistance are known, each associated with different ligase genes, *(vanA, vanB, vanC1,* and *vanC2)*, and a fourth type, VanD, has been reported recently (25). VanA and VanB type resistance is encoded by gene clusters that are acquired (i.e., not part of the normal genome of enterococci) and are often transferable. In contrast, *vanC1* and *vanC2* are normally occurring genes that are endogenous species characteristics of *E. gallinarum* and *E. casseliflavus*, respectively, and are not transferable. The acquired gene clusters associated with *vanA* and *vanB* are found in different genetic surroundings. The *vanA* gene cluster has been found in a small Tn*3*-like transposon, Tn*1546*, and in elements that appear to be closely related (e.g., Tn*5488*, which has an insertion sequence [IS*1251*] within Tn*1546* [48,49]) or lacking *vanZ* (50). These elements have in turn been found on both transferable and nontransferable plasmids, as well as on the chromosome of the host strain. VanB type resistance was initially not found to be transferable, but at least in some instances, the *vanB* gene cluster has been found on large (90 kb to 250 kb) chromosomally located transferable elements, one of which contains within it a 64-kb composite transposon (Tn*1547*) (Figure 2; 14). More recently, *vanB* has been found as part of plasmids.

In addition to being found in different genetic surroundings, the *vanA* and *vanB* gene clusters have also been found in a number of different bacterial species. *vanA* has been found in multiple enterococcal species as well as in lactococci, *Orskovia*, and *Arcanobacteria* (51). The distribution of the *vanB* gene cluster seems somewhat more restricted, having been found primarily in *E. faecium* and *E. faecalis*, although it has recently been found in *Streptococcus bovis* (52).

When vancomycin-resistant enterococci (VRE) from patients in a given hospital have been examined, particularly after the first recovery of VRE, evidence is often found of a single or predominant strain (53-58). Finding isolates with identical or highly related PFGE patterns in different hospitals indicates interhospital clonal transmission (59-61). Some reports do not find a single or a predominant strain, especially when VRE have been present in a hospital or area for some time (62,63). This was also true of two reports from France in which all of 16 and all of 24 vancomycin-resistant *E. faecium* were different (50,64,65). The reports from France likely reflect another observation regarding the diversity of vancomycin resistance. v*anA* has also been shown to be present in normal fecal enterococci of healthy, nonhospitalized persons in different parts of Europe (66-68); in one study, 20 different strains (identified by PFGE) of vancomycin-resistant *E. faecium* were found in the fecal flora of 17 persons in two areas in Belgium (68). *vanA* containing *E. faecium* have also been found in the feces of healthy animals as well as from animal products in Europe (50,67,69-71); in one study, VRE were found in the feces of healthy meat-eaters but not vegetarians (72). VRE have not, however, been found as part of the normal fecal flora in the United States (73) possibly because glycopeptides, often used in animal feed in Europe, are not used in the United States. For example, 24,000 kg annually of the glycopeptide avoparcin were reportedly used in recent years in Denmark (74). Reports of VRE in the feces of animals on farms using glycopeptides, but not in those without such use, support this hypothesis (71,75). While oral glycopeptide use markedly increases the numbers of VRE per gram of stool in humans (68) and by analogy, presumably does so in animals, glycopeptide use does not explain the origin of these gene clusters.

The problem of multidrug-resistant enterococci promises to be with us for the foreseeable future. The enterococcus has likely emerged as a major nosocomial pathogen in part because of its resistance to multiple antibiotics, which allows it to survive and subsequently infect patients. With its propensity to acquire new traits, such as high-level gentamicin, penicillin, and vancomycin resistance, the enterococcus continues to create new therapeutic problems and dilemmas; its ability to transfer some of its plasmids to streptococci and staphylococci and the implications of a possible spread of penicillin and vancomycin resistance to these and other gram-positive species are also of concern.

Dr. Murray is professor and director, Division of Infectious Diseases, and codirector, Center for the Study of Emerging and Re-emerging Pathogens, University of Texas Houston-Medical School. Her main interests are in the areas of antibiotic resistance, molecular epidemiology, and bacterial pathogenesis. Recent work has been focused on possible mechanisms of virulence of enterococci.

### References

1. Murray BE. The life and times of the enterococcus. Clin Microbiol Rev 1990;3:46-65.
2. Moellering RC Jr. Emergence of enterococcus as a significant pathogen. Clin Infect Dis 1992;14:1173-8.
3. Schaberg DR, Culver DH, Gaynes RP. Major trends in the microbial etiology of nosocomial infection. Am J Med 1991;91(Suppl 3B):72S-75S.
4. Hodges TL, Zighelboim-Daum S, Eliopoulos GM, Wennersten C, Moellering RC Jr. Antimicrobial susceptibility changes in *Enterococcus faecalis* following various penicillin exposure regimens. Antimicrob Agents Chemother 1992;36:121-5.
5. Zervos MJ, Schaberg DS. Reversal of the *in vitro* susceptibility of enterococci to trimethoprim-sulfamethoxazole by folinic acid. Antimicrob Agents Chemother 1985;28:446-8.
6. Najjar A, Murray BE. Failure to demonstrate a consistent *in vitro* bactericidal effect of trimethoprim-sulfamethoxazole against enterococci. Antimicrob Agents Chemother 1987;31:808-10.
7. Chenoweth CE, Robinson KA, Schaberg DR. Efficacy of ampicillin versus trimethprim-sulfamethoxazole in a mouse model of lethal enterococcal peritonitis. Antimicrob Agents Chemother 1990;34:1800-2.
8. Grayson ML, Thauvin-Eliopoulos C, Eliopoulos GM, Yao JDC, DeAngelis DV, Walton L, et al. Failure of trimethoprim-sulfamethoxazole therapy in experimental enterococcal endocarditis. Antimicrob Agents Chemother 1990;34:1792-4.
9. Clewell DB, Keith EW. Sex pheromones and plasmid transfer in *Enterococcus faecalis*. Plasmid 1989;21:175-84.
10. Clewell DB. Plasmids, drug resistance, and gene transfer in the genus *Streptococcus*. Microbiol Rev 1981;45:409-36.
11. Clewell DB. Conjugative transposons and the dissemination of antibiotic resistance in streptococci. Annu Rev Microbiol 1986;40:635-59.
12. Roberts MC. Characterization of the Tet M determinants in urogenital and respiratory bacteria. Antimicrob Agents Chemother 1990;34:476-8.
13. Roberts MC, Hillier SL. Genetic basis of tetracycline resistance in urogenital bacteria. Antimicrob Agents Chemother 1990;34:261-4.
14. Quintiliani R Jr, Courvalin P. Characterization of Tn*1547*, a composite transposon flanked by the IS*16* and IS*256*-like elements, that confers vancomycin resistance in *Enterococcus faecalis* BM4281. Gene 1996;172:1-8.
15. Ferretti JJ, Gilmore KS, Courvalin P. Nucleotide sequence analysis of the gene specifying the bifunctional 6'-aminoglycoside acetyltransferase 2"-aminoglycoside phosphotransferase enzyme in *Streptococcus faecalis* and identification and cloning of gene regions specifying the two activities. J Bacteriol 1986;167:631-8.
16. Carlier C, Courvalin P. Emergence of 4',4"-aminoglycoside nucleotidyltransferase in enterococci. Antimicrob Agents Chemother 1990;34:1565-9.
17. Chow JW, Zervos MJ, Lerner SA, Thal LA, Donabedian SM, Jaworski DD, et al. A novel gentamicin resistance gene in *Enterococcus*. Antimicrob Agents Chemother 1997;41:511-4.

18. Murray BE. ß-lactamase-producing enterococci. Antimicrob Agents Chemother 1992;36:2355-9.
19. Murray BE, Singh KV, Markowitz SM, Lopardo HA, Patterson JE, Zervos MJ, et al. Evidence for clonal spread of a single strain of ß-lactamase-producing *Enterococcus faecalis* to six hospitals in five states. J Infect Dis 1991;163:780-5.
20. Rhinehart E, Smith NE, Wennersten C, Gorss E, Freeman J, Eliopoulos GM, et al. Rapid dissemination of ß-lactamase-producing, aminoglycoside-resistant *Enterococcus faecalis* among patients and staff on an infant-toddler surgical ward. N Engl J Med 1990;323:1814-8.
21. Wells VD, Wong ES, Murray BE, Coudron PE, Williams DS, Markowitz DM. Infections due to beta-lactamase-producing, high-level gentamicin-resistant *Enterococcus faecalis*. Ann Intern Med 1992;116:285-92.
22. Grayson ML, Eliopoulos GM, Wennersten CB, Ruoff KL, de Girolami PC, Ferraro M-J, Moellering, RC Jr. Increasing resistance to ß-lactam antibiotics among clinical isolates of *Enterococcus faecium*: a 22-year review at one institution. Antimicrob Agents Chemother 1991;35:2180-4.
23. Fontana R, Ligozzi M, Pittaluga F, Satta G. Intrinsic penicillin resistance in enterococci. Microbial Drug Resist 1996;2:209-13.
24. Rice LB, Carias LL, Rudin SB, Donskey CJ. Abstract #LB-17 Transferable ampicillin resistance in *Enterococcus faecium* and linkage of ampicillin and vancomycin-resistance determinants [abstract]. In program of the 37th ICAAC Program Addendum; 1997 Sep 28–Oct1; Toronto, Ontario, Canada.
25. Perichon B, Reynolds P, Courvalin P. VanD-type glycopeptide-resistant *Enterococcus faecium* BM4339. Antimicrob Agents Chemother 1997;41:2016-8.
26. Derlot E, Courvalin P. Mechanisms and implications of glycopeptide resistance in enterococci. Am J Med 1991;91(Suppl 3B):82S-5S.
27. Arthur M, Molinas C, Bugg TDH, Wright GD, Walsh CT, Courvalin P. Evidence for *in vivo* incorporation of D-lactate into peptidoglycan precursors of vancomycin-resistant enterococci. Antimicrob Agents Chemother 1992;36:867-9.
28. Dutka-Malen S, Molinas C, Arthur M, Courvalin P. Sequence of the *vanC* gene of *Enterococcus gallinarum* BM4174 encoding a D-alanine:D-alanine ligase-related protein necessary for vancomycin resistance. Gene 1992;112:53-8.
29. Navarro F, Courvalin P. Analysis of genes encoding D-alanine-D-alanine ligase-related enzymes in *Enterococcus casseliflavus* and *Enterococcus flavescens*. Antimicrob Agents Chemother 1994;38:1788-93.
30. Billot-Klein D, Gutmann L, Sable S, Guittet E, van Heijenoort J. Modification of peptidoglycan precursors is a common feature of the low-level vancomycin-resistant VANB-type enterococcus D366 and of the naturally glycopeptide-resistant species *Lactobacillus casei, Pediococcus pentosaceus, Leuconostoc mesenteroides,* and *Enterococcus gallinarum*. J Bacteriol 1994;176:2398-405.
31. Moellering RC Jr, Wennersten C, Medrek T, Weinberg AN. 1971. Prevalence of high-level resistance to aminoglycosides in clinical isolates of enterococci. Antimicrobial agents and chemotherapy—1970. Washington: American Society for Microbiology; 1971. p. 335-40.
32. Watanakunakorn C. The prevalence of high-level aminoglycoside resistance among enterococci isolated from blood cultures during 1980-1988. J Antimicrob Chemother 1989;24:63-8.
33. Phillips I, King A, Gransden WR, Eykyn SJ. The antibiotic sensitivity of bacteria isolated from the blood of patients in St. Thomas' Hospital, 1969-1988. J Antimicrob Chemother 1990;25:59-80.
34. Zervos MJ, Dembinski S, Mikesell T, Schaberg DR. High-level resistance to gentamicin in *Streptococcus faecalis*: risk factors and evidence for exogenous acquisition of infection. J Infect Dis 1986;153:1075-83.
35. Eliopoulos GM, Wennersten C, Zighelboim-Daum S, Reiszner E, Goldmann D, Moellering RC Jr. High-level resistance to gentamicin in clinical isolates of *Streptococcus (Enterococcus) faecium*. Antimicrob Agents Chemother 1988;32:1528-32.
36. Murray BE, Singh KV, Heath JD, Sharma BR, Weinstock GM. Comparison of genomic DNAs of different enterococcal isolates using restriction endonucleases with infrequent recognition sites. J Clin Microbiol 1990;28:2059-63.
37. Zervos MJ, Mikesell TS, Schaberg DR. Heterogeneity of plasmids determining high-level resistance to gentamicin in clinical isolates of *Streptococcus faecalis*. Antimicrob Agents Chemother 1986;30:78-81.
38. Hodel-Christian SL, Murray BE. Characterization of the gentamicin resistance transposon Tn*5281* from *Enterococcus faecalis* and comparison to staphylococcal transposons Tn*4001* and Tn*4031*. Antimicrob Agents Chemother 1991;35:1147-52.
39. Hodel-Christian SL, Smith M, Zscheck KZ, Murray BE. 1991. Comparison of a gentamicin resistance transposon and a beta-lactamase gene from enterococci to those from staphylococci. In: Dunny GM, Cleary PP, McKay LL, editors. Genetics and molecular biology of streptococci, lactococci, and enterococci. Washington: American Society for Microbiology; 1991. p. 54-8.
40. Thal LA, Chow JW, Clewell DB, Zervos MJ. Tn*924*, a chromosome-borne transposon encoding high-level gentamicin resistance in *Enterococcus faecalis*. Antimicrob Agents Chemother 1994;38:1152-6.
41. Rice LB, Carias LL, Marshall SH, Bonafede ME. 1996. Sequences found on staphylococcal ß-lactamase plasmids integrated into the chromosome of *Enterococcus faecalis* CH116. Plasmid 1996;35:81-90.
42. Seetulsingh PS, Tomayko JF, Coudron PE, Markowitz SM, Skinner C, Singh KV, Murray BE. Chromosomal DNA restriction endonuclease digestion patterns of ß-lactamase-producing *Enterococcus faecalis* isolates collected from a single hospital over a 7-year period. J Clin Microbiol 1996;34:1892-6.
43. Tomayko JF, Murray BE. Analysis of *Enterococcus faecalis* isolates from intercontinental sources by multilocus enzyme electrophoresis and pulsed-field gel electrophoresis. J Clin Microbiol 1995;33:2903-7.
44. Coudron PE, Markowitz SM, Wong ES. Isolation of a ß-lactamase-producing, aminoglycoside-resistant strain of *Enterococcus faecium*. Antimicrob Agents Chemother 1992;36:1125-6.
45. Patterson JE, Singh KV, Murray BE. Epidemiology of an endemic strain of ß-lactamase-producing *Enterococcus faecalis*. J Clin Microbiol 1991;29:2513-6.

46. Handwerger S, Perlman DC, Altarac D, McAuliffe V. Concomitant high-level vancomycin and penicillin resistance in clinical isolates of enterococci. Clin Infect Dis 1992;14:655-61.
47. Miranda AG, Singh KV, Murray BE. DNA fingerprinting of *Enterococcus faecium* by pulsed-field gel electrophoresis may be a useful epidemiologic tool. J Clin Microbiol 1991;29:2752-7.
48. Handwerger S, Skoble J. Identification of chromosomal mobile element conferring high-level vancomycin resistance in *Enterococcus faecium*. Antimicrob Agents Chemother 1995;39:2446-53.
49. Handwerger S, Skoble J, Discotto LF, Pucci MJ. Heterogeneity of the *vanA* gene cluster in clinical isolates of enterococci from the Northeastern United States. Antimicrob Agents Chemother 1995;39:362-8.
50. van den Bogaard AE, Jensen LB, Stobberingh EE. Vancomycin-resistant enterococci in turkeys and farmers. N Engl J Med 1997;337:1558-9.
51. Power EGM, Abdulla YH, Talsania HG, Spice W, Aathithan S, French GL. *vanA* genes in vancomycin-resistant clinical isolates of *Oerskovia turbata* and *Arcanobacterium (Corynebacterium) haemolyticum*. J Antimicrob Chemother 1995;36:595-606.
52. Poyart C, Pierre C, Quesne G, Pron B, Berche P, Trieu-Cuot P. Emergence of vancomycin resistance in the genus *Streptococcus*: characterization of a *vanB* transferable determinant in *Streptococcus bovis*. Antimicrob Agents Chemother 1997;41:24-9.
53. Biavasco F, Miele A, Vignaroli C, Manso E, Lupid R, Varaldo PE. Genotypic characterization of a nosocomial outbreak of VanA *Enterococcus faecalis*. Microb Drug Resist 1996;2:231-7.
54. Boyce JM, Opal SM, Chow JW, Zervos MJ, Potter-Bynoe G, Sherman CB, et al. Outbreak of multidrug-resistant *Enterococcus faecium* with transferable *vanB* class vancomycin resistance. J Clin Microbiol 1994;32:1148-53.
55. Handwerger S, Raucher B, Altarac D, Monka J, Marchione S, Singh KV, et al. Nosocomial outbreak due to *Enterococcus faecium* highly resistant to vancomycin, penicillin and gentamicin. Clin Infect Dis 1993;16:750-5.
56. Livornese LL Jr, Dias S, Samel C, Romanowski B, Taylor S, May P, et al. Hospital-acquired infection with vancomycin-resistant *Enterococcus faecium* transmitted by electronic thermometers. Ann Intern Med 1992;117:112-6.
57. Moreno F, Grota P, Crisp C, Magnon K, Melcher GP, Jorgensen JH, Patterson JE. Clinical and molecular epidemiology of vancomycin-resistant *Enterococcus faecium* during its emergence in a city in southern Texas. Clin Infect Dis 1995;21:1234-7.
58. Perlada DE, Smulian AG, Cushion MT. Molecular epidemiology and antibiotic susceptibility of enterococci in Cincinnati, Ohio: a prospective citywide survey. J Clin Microbiol 1997;35:2342-7.
59. Chow JW, Kuritza A, Shlaes DM, Green M, Sahm DF, Zervos MJ. Clonal spread of vancomycin-resistant *Enterococcus faecium* between patients in three hosptials in two states. J Clin Microbiol 1993;31:1609-11.
60. Clark NC, Cooksey RC, Hill BC, Swenson JM, Tenover FC. Characterization of glycopeptide-resistant enterococci from U.S. hospitals. Antimicrob Agents Chemother 1993;37:2311-7.
61. Sader HS, Pfaller MA, Tenover FC, Hollis RJ, Jones RN. Evaluation and characterization of multiresistant *Enterococcus faecium* from 12 U.S. medical centers. J Clin Microbiol 1994;32:2840-2.
62. Boyle JF, Soumakis SA, Rendo A, Herrington JA, Gianarkis DG, Thurberg BE, Painter BG. Epidemiologic analysis and genotypic characterization of a nosocomial outbreak of vancomycin-resistant enterococci. J Clin Microbiol 1993;31:1280-5.
63. Morris JG Jr, Shay DK, Hebden JN, McCarter RJ Jr, Perdue BE, Jarvis W, et al. Enterococci resistant to multiple antimicrobial agents, including vancomycin. Ann Intern Med 1995;123:250-9.
64. Bingen EH, Denamur E, Lambert-Zechovsky NY, Elion J. Evidence for the genetic unrelatedness of nosocomial vancomycin-resistant *Enterococcus faecium* strains in a pediatric hospital. J Clin Microbiol 1991;29:1888-92.
65. Plessis P, Lamy T, Donnio PY, Autuly F, Grulois I, LePrise PY, Avril JL. Epidemiologic analysis of glycopeptide-resistant *Enterococcus* strains in neutropenic patients receiving prolonged vancomycin administration. Eur J Clin Microbiol Infect Dis 1995;14:959-63.
66. Jordens JZ, Bates J, Griffiths DT. Faecal carriage and nosocomial spread of vancomycin-resistant *Enterococcus faecium*. J Antimicrob Chemother 1994;34:515-28.
67. Klare I, Heier H, Claus H, Bohme G, Marin S, Seltmann G, et al. *Enterococcus faecium* strains with *vanA*-mediated high-level glycopeptide resistance isolated from animal foodstuffs and fecal samples of humans in the community. Microbial Drug Resist 1995;3:265-72.
68. Van der Auwera P, Pensart N, Korten V, Murray BE, Leclercq R. Influence of oral glycopeptides on the fecal flora of human volunteers: selection of highly-glycopeptide-resistant enterococci. J Infect Dis 1995;173:1129-36.
69. Aarestrup FM, Ahrens P, Madsen M, Pallesen LV, Poulsen RL, Westh H. Glycopeptide susceptibility among Danish *Enterococcus faecium* and *Enterococcus faecalis* isolates of animal and human origin and PCR identification of genes within the VanA cluster. Antimicrob Agents Chemother 1996;40:1938-40.
70. Bates J, Jordens Z, Selkon JB. Evidence for an animal origin of vancomycin-resistant enterococci [letter]. Lancet 1993;342:490.
71. Klare I, Heier H, Claus H, Reissbrodt R, Witte W. *vanA*-mediated high-level glycopeptide resistance in *Enterococcus faecium* from animal husbandry. FEMS Microbiol Lett 1995;125:165-72.
72. Schouten MA, Voss A, Hoogkamp-Korstanje JAA. VRE and meat. Lancet 1997;349:1258.
73. Coque TM, Tomayko JF, Ricke SC, Okhuysen PO, Murray BE. Vancomycin-resistant enterococci from nosocomial, community, and animal sources in the United States. Antimicrob Agents Chemother 1996;40:2605-9.

74. Kjerulf A, Pallesen L, Westh H. Vancomycin-resistant enterococci at a large university hospital in Denmark. APMIS 1996;104:475-9.

75. Klare I, Heier H, Claus H, Witte W. Environmental strains of *Enterococcus faecium* with inducible high-level resistance to glycopeptides. FEMS Microbiol Lett 1993;106:23-30.
